# Detection of *BRAF* mutation in Chinese tumor patients using a highly sensitive antibody immunohistochemistry assay

**DOI:** 10.1038/srep09211

**Published:** 2015-03-18

**Authors:** Tian Qiu, Haizhen Lu, Lei Guo, Wenting Huang, Yun Ling, Ling Shan, Wenbin Li, Jianming Ying, Ning Lv

**Affiliations:** 1 Department of Pathology, Cancer Hospital, Peking Union Medical College and Chinese Academy of Medical Sciences, Beijing, China

## Abstract

*BRAF* mutations can be found in various solid tumors. But accurate and reliable screening for *BRAF* mutation that is compatible for clinical application is not yet available. In this study, we used an automated immunohistochemistry (IHC) staining coupled with mouse monoclonal anti-BRAF V600E (VE1) primary antibody to screen the *BRAF* V600E mutation in 779 tumor cases, including 611 colorectal carcinomas (CRC), 127 papillary thyroid carcinomas (PTC) and 41 malignant melanomas. Among the 779 cases, 150 cases were positive for BRAF (V600E) staining, including 38 (of 611, 6%) CRCs, 102 (of 127, 80%) PTCs and 10 (of 41, 24%) malignant melanomas. Sanger sequencing and real-time PCR confirmed the sensitivity and specificity of IHC staining for the V600E mutation are 100% and 99%, respectively. Therefore, our study demonstrates that the fully automated IHC is a reliable tool to determine *BRAF* mutation status in CRC, PTC and melanoma and can be used for routine clinical screen.

The v-Raf murine sarcoma viral oncogene homolog B1 (*BRAF*) gene encodes a serine/threonine protein kinase that is belonged to the Mitogen Activated Protein Kinase cascade (MAPK) signaling pathway. Since the discovery in 2002, *BRAF* mutations have been found in various solid tumors, including thyroid carcinoma, malignant melanoma and colorectal carcinoma^1^. The most common *BRAF* mutation is the T1799A transversion, which results the substitution of glutamic acid for valine at amino acid 600 (V600E) and leads to constitutive activation of BRAF^2^,^3^,^4^.

Due to poor response to convention chemotherapy, melanoma has a poor prognosis. Recent development of Vemurafenib that specifically targets BRAF(V600E) mutation have yield promising results^5^. The diagnostic test that is able to recognize melanoma patients harboring mutant *BRAF* allow the identification of patients who can benefit from Vemurafenib treatment^6^,^7^,^8^,^9^,^10^. BRAF is also important in the development of colorectal carcinoma (CRC). The progression of CRC relies on oncogenic activation of signaling pathways downstream of the EGFR, including *BRAF* mutation^11^,^12^,^13^. Papillary thyroid carcinoma (PTC) is the most common type of thyroid carcinoma, accounting for more than 80% of the thyroid carcinoma. Many works have shown that a high prevalence of *BRAF* mutations was found in PTC^14^,^15^. The rate of *BRAF* mutation increased significantly over a 15-year period^16^.

Currently, Sanger sequencing and real-time PCR are the clinical methods that are used to detect *BRAF* mutations in diagnostic laboratories, including selecting melanoma patients eligible to Vemurafenib treatment. However, Sanger sequencing and real-time PCR have significant disadvantages. Both methods are expensive and time-consuming, which limited their clinical application. Immunohistochemistry (IHC) is a technique that is readily available in pathology laboratories, and it is relatively cheap, efficient and suitable as a screening tool. Recently, several studies have demonstrated that a BRAF V600E mutation–specific monoclonal antibody (clone VE1) could detect the V600E mutated BRAF protein in different carcinomas. Yet some researchers believe that IHC is not a valid surrogate for sequencing to detect V600E mutated BRAF in CRC^17^,^18^,^19^,^20^. Hence, the optimal method to detect *BRAF* mutations in cancers remains to be determined. Here we report a novel and fully automated IHC assay to screen the *BRAF* V600E mutation in Chinese patients with CRC, PTC and melanoma. The sensitivity and specifity of this novel IHC assay are 100% and 99% respectively when compared with Sanger sequencing and real-time PCR for the detection of *BRAF V600E* mutation.

## Results

### Immunohistochemistry

**Figure 1:**
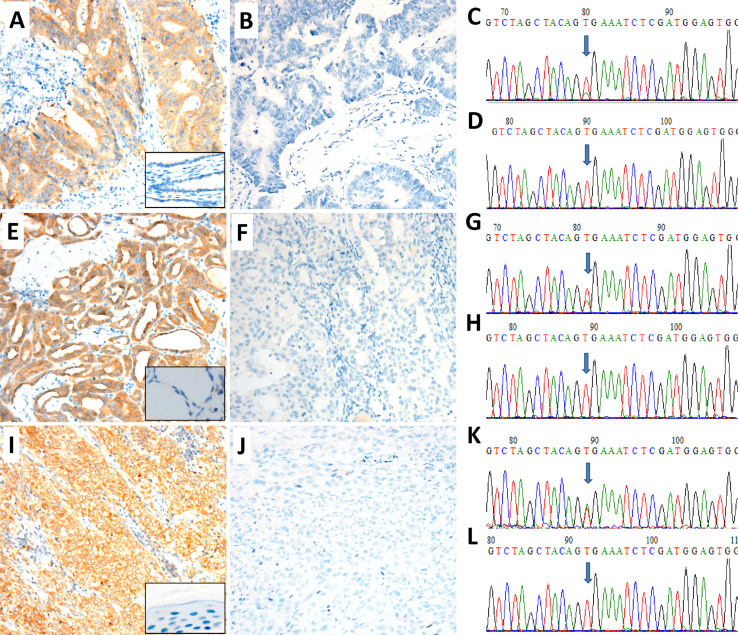
Detection of *BRAF* mutation in colorectal carcinoma (CRC), papillary thyroid carcinoma (PTC) and melanoma by immunochemistry (IHC) and Sanger sequencing. Representative images of positive (A, E, I) and negative (B, F, J) for BRAF expression by VE1 IHC. Boxes in A, E, I show the negative controls from their corresponding non-tumor tissues. C, G and K images show a c.1799T > A (p.V600E) point mutation (arrow) of the *BRAF* gene. D, H and L images show the *BRAF* mutation (V600E) negative. BRAF Ventana VE1 IHC assay revealed strong expression in *BRAF* mutation positive patients and no expression in *BRAF* mutation negative patients in colorectal carcinoma (A–D), papillary thyroid carcinoma (E–H) and melanoma (I–L), respectively. Original magnification ×200.

Ventana IHC assay using BRAF V600E (VE1) mouse monoclonal primary antibody was performed to screen for the *BRAF* V600E mutation in 779 patients, including 611 cases of CRC, 127 cases of PTC and 41 cases of malignant melanoma. Among the 779 cases, 150 cases were positive for BRAF (V600E) staining, including 38 cases (of 611, 6%) of CRC, 102 (of 127, 80%) cases of PTC and 10 (of 41, 24%) cases of malignant melanoma (Figure 1).

### Molecular analyses

A total of 349 patients were analyzed for *BRAF* mutation by both Sanger sequencing and real-time PCR (Cobas 4800 BRAF V600 Mutation Test), including 181 cases of CRC, 127 cases of PTC and 41 cases of malignant melanoma. Of the 349 tumors, 148 harbored T1799A mutation (p.V600E) of the *BRAF* gene by both Sanger sequencing and Cobas 4800 BRAF V600 Mutation Test, including 38 cases of CRC, 100 cases of PTC and 10 cases of malignant melanoma (Figure 1). No other mutation beyond V600E were detected in the exon 15 of *BRAF* gene. The results of Sanger sequencing and Cobas 4800 BRAF V600 Mutation Test matched to each other in all tested tumors (Table 1).

### Comparison of immunohistochemistry and molecular analyses

As shown in Table 2, 150 patients with Ventana IHC *BRAF* V600E mutation were positive and 148 patients were positive by molecular assays. All patients with Ventana IHC *BRAF* V600E mutation negative were negative by molecular genetic techniques. Two PTCs with *BRAF* mutation positive by Ventana IHC were found to be negative by molecular assays. The details of these two discrepant cases for *BRAF* mutation detection were shown in Table 3. The sensitivity of BRAF Ventana IHC was measured as the proportion of the IHC positive cases in the molecular assays positive cases (148/148). The specificity of BRAF Ventana IHC was determined as the proportion of the IHC negative cases in the molecular assays negative cases (199/201). Therefore, the sensitivity and specificity of BRAF Ventana IHC for *BRAF V600E* mutation detection were 100% and 99%, respectively.

## Discussion

In this study, we have performed a fully automated IHC analysis to detect the V600E mutated BRAF protein using the Ventana BRAF V600E (VE1) mouse monoclonal primary antibody combined with the Optiview DAB IHC detection kit and compared it on matched samples with conventional molecular methods. Using Sanger sequencing and real-time PCR results as the reference, the sensitivity and specificity of VE1 immunohistochemistry for the *BRAF V600E* mutation are 100% and 99%, respectively. The BRAF Ventana IHC assay is a fully automated IHC test that can provide laboratory professionals and pathologists with a sensitive and specific standardized test for *BRAF* V600E mutation in formalin fixed paraffin embedded (FFPE) tissues. The interpretation of the results is clear. The negative and positive samples can be easily distinguished without the need of a subjective IHC scoring system based on staining intensity or percentage of positively stained cells.

The *BRAF* mutation is a promising diagnostic and prognostic marker and is also an important indicator for targeted therapy by BRAF V600E specific inhibitors. Accurate and reliable screening for *BRAF* mutation would be highly desired. In this study, we screened *BRAF* mutation status by Sanger sequencing, real-time PCR and immunohistochemistry. The Cobas 4800 BRAF V600 Mutation Test is the FDA-approved companion diagnostic for Vemurafenib, which consists of a real-time quantitative PCR step with two primers that amplify a 116 base pair fragment of the exon 15 of *BRAF* (containing codon 600). However, the real-time PCR cannot identify all *BRAF* mutation types. It can detect the main mutation types. Though it is designed to detect *BRAF* V600E (c.1799T > A) mutation, this kit also has some degree of cross-reactivity with V600K (c.1798_1799GT > AA) and other less common mutations such as V600E2 (c.1799_1800TG > AA), V600R (c.1798_1799GT > AG) and V600D (c.1799_1800TG > AC)^21^. Usually the sensitivity of the real-time PCR is higher than Sanger sequencing, although Sanger sequencing can detect all types of *BRAF* mutation. Discrepancies between these two assays have been previously reported. However, in our study, the results of *BRAF* mutation from the Sanger sequencing and Cobas 4800 BRAF V600 Mutation Test 100% correlates to each other, and no discrepancies were observed. The main reason for this high concordance might be the quality control of tumor samples. The H&E slides of all tumor tissues were evaluated for tumor content before proceeding to molecular analysis. When the proportion of tumor cells was less than 50%, the slides were marked for subsequent tumor macrodissection to enrich tumor cell populations before testing. Immunohistochemistry is a simple, rapid and relatively inexpensive method for detection of *BRAF* mutation. We performed immunohistochemistry using the Ventana BRAF V600E (VE1) mouse monoclonal primary antibody on Ventana Benchmark IHC automated strainers in combination with the OptiView DAB IHC detection kit in CRC, PTC and melanoma samples, and the results show highly sensitivity (100%) and specificity (99%). Our results demonstrated BRAF IHC is a simple, accurate and reliable screening method, which can be used routinely for *BRAF* mutation analyses in clinics.

Most importantly, the present study has used the fully automated IHC method to detect *BRAF* V600E mutation in various carcinomas including CRC, PTC and melanoma. Recently published studies exploring the VE1 antibody have also shown a high specificity and sensitivity, but only focus on single tumor type or small sample size^22^,^23^,^24^. In this study, we screen the *BRAF* V600E mutation in 779 patients, including 611 CRCs, 127 PTCs and 41 malignant melanoma. Of the 779 cases, 349 cases were also independently screened by Sanger sequencing and Cobas 4800 BRAF V600 Mutation Test, including 181 CRCs, 127 PTCs and 41 malignant melanomas. Only 2 PTCs with *BRAF* mutation positive by VE1 IHC were found to be negative by molecular genetic techniques. Although molecular genetic techniques are the standard method to detect *BRAF* mutations, it requires high tumor contents, special equipment and skilled operator. Our study demonstrated that IHC would be a useful tool for *BRAF* mutation screening in CRC, PTC and melanoma and potentially other tumor types.

## Methods

### Patients

The study was approved by the medical ethical committee of Cancer Institute and Hospital, Chinese Academy of Medical Sciences. The methods were carried out in accordance with the approved guidelines. The informed consents were obtained from all patients. A total of 779 patients were enrolled in this study, including 611 cases of CRC, 127 cases of PTC and 41 cases of malignant melanoma. All patients were treated at the Cancer Hospital, Chinese Academy of Medical Sciences, from July 2010 to June 2014. A total of 181 CRC samples and all cases of PTC and melanoma screened for *BRAF* mutation by IHC were also analyzed by both molecular methods. All cases were formalin-fixed and paraffin-embedded specimens. A pathologist evaluated the H&E slides for tumor content to make sure the proportion of tumor cells was more than 50%, otherwise, the slides were marked for subsequent tumor dissection to enrich tumor cell populations before proceeding to molecular analysis.

### Immunohistochemistry

IHC for BRAF protein expression was performed on 4 μm-thick sections of formalin-fixed, paraffin-embedded tissues, using the Ventana BRAF V600E (VE1) Mouse Monoclonal Primary Antibody on Ventana Benchmark IHC automated slide strainer in combination with the OptiView DAB IHC detection kit. The specimens were fixed in 10% neutral buffered formalin for 24–48 hours. Negative and positive controls were included in each round of analysis. Briefly, after deparaffinization, the slides were pretreated with cell conditioning 1 for 64 minutes for antigen unmasking and followed by pre-primary antibody peroxidase inhibition. The slides were then incubated with the VE1 antibody at 37°C for 16 minutes, and counterstained with hematoxylin II for 4 minutes and bluing reagent for 4 minutes. The staining pattern for anti-BRAF V600E (VE1) antibody is cytoplasmic staining of tumor cells. The cases of cytoplasmic staining in tumor cells are positive when the anti-BRAF V600E (VE1) antibody is used and no staining when the negative control is selected.

### Sanger Sequencing

Genomic DNA was extracted from paraffin-embedded tumor tissues using QIAamp® DNA Mini Kit (Qiagen, Germany), according to the manufacturer's instructions. Quality and concentration of the DNA samples were examined by NanoDrop (Thermo). The primers used to amplify *BRAF* exon 15 were as follows: forward 5′-TCATAATGCTTGCTCTGATAGGA-3′ and reverse 5′-GGCCAAAAATTTAATCAGTGGA-3′. Polymerase chain reaction (PCR) was carried as following: a final volume of 25 μl containing purified genomic DNA (100 ng/μl) 1 μl, 10 × ABI buffer 2.5 μl, MgCl_2_ (25 mM) 1.5 μl, dNTP (2.5 mM) 2 μl, ABI AmpliTaq Gold DNA Taq polymerase 0.125 μl (5 U/μl), forward primer and reverse primer (10 μM) 1 μl, after denaturation at 95°C for 10 minutes, 38 amplification cycles at 95°C for 30 s, 56°C for 30 s, 72°C for 45 s, and elongation at 72°C for 10 minutes. The PCR products sequencing were performed with ABI BigDye Terminator v3.1 Cycle Sequencing Kit (Applied Biosystems) according to the manufacturer's instructions. The sequencing primers were the same as the PCR primers. Sequencing reactions were electrophoresed on an ABI 3500XL genetic analyzer (Applied Biosystems). Sequence data were analyzed using an ABI 3500XL DNA Analyzer (Applied Biosystems).

### Real-time PCR

The real-time PCR was performed using the Cobas 4800 BRAF V600 Mutation Test Kit. DNA was adjusted to a fixed concentration and added to the detection mixture. The target DNA was then amplified and detected on the Cobas z 480 analyzer using the amplification and detection reagents provided in the Cobas 4800 BRAF V600 Mutation Test Kit. Tests follow the Cobas 4800 system Operator's Manual Software Version 2.0 for Cobas 4800 BRAF V600 Mutation Test for detailed instructions for the BRAF workflow steps. All runs and specimen validation were performed by the Cobas 4800 software.

## Author Contributions

T.Q., J.Y. and N.L. designed experiments, T.Q. and J.Y. conducted experiments and data analysis, and wrote the paper. H.L. and W.H. performed the pathological diagnosis. T.Q., L.G., W.H., Y.L., L.S. and W.L. performed the experiments. All authors reviewed the manuscript.

## Figures and Tables

**Table 1:** Correlation of *BRAF* mutation detection between Sanger sequencing and real-time PCR

		real-time PCR		
		positive	negative	Total
Sanger sequencing	positive	148	0	148 (42%)
	negative	0	201	201 (58%)
Total		148 (42%)	201 (58%)	349 (100%)

BRAF real-time PCR using Cobas 4800 BRAF V600 Mutation Test.

**Table 2:** Correlation of *BRAF* mutation detection between IHC and molecular assays

		BRAF molecular assays		
		positive	negative	Total
IHC	positive	148	2	150 (43%)
	negative	0	199	199 (57%)
Total		148 (42%)	201 (58%)	349 (100%)

IHC, immunohistochemistry; BRAF molecular assays using Sanger sequencing and Cobas 4800 BRAF V600 Mutation Test.

**Table 3:** List of discrepant cases for *BRAF* mutation detection between IHC and molecular assays

Sample ID	Sex	Age	Histologic diagnosis	IHC	molecular assays
256	F	50	Papillary thyroid carcinoma	Positive	Negative
307	F	36	Papillary thyroid carcinoma	Positive	Negative

## References

[b1] Davies H. *et al.* Mutations of the BRAF gene in human cancer. Nature 417, 949–954 (2002). 12068308 10.1038/nature00766

[b2] Cohen Y. *et al.* BRAF mutation in papillary thyroid carcinoma. J Natl Cancer Inst 95, 625–627 (2003). 12697856 10.1093/jnci/95.8.625

[b3] Dhomen N. & Marais R. BRAF signaling and targeted therapies in melanoma. Hematol Oncol Clin North Am 23, 529–545 (2009). 19464601 10.1016/j.hoc.2009.04.001

[b4] Ilie M. *et al.* Diagnostic value of immunohistochemistry for the detection of the BRAFV600E mutation in primary lung adenocarcinoma Caucasian patients. Ann Oncol 24, 742–748 (2013). 23131393 10.1093/annonc/mds534

[b5] Ascierto P. A. *et al.* The role of BRAF V600 mutation in melanoma. J Transl Med 10, 85 (2012). 22554099 10.1186/1479-5876-10-85 PMC3391993

[b6] Greaves W. O. *et al.* Frequency and spectrum of BRAF mutations in a retrospective, single-institution study of 1112 cases of melanoma. J Mol Diagn 15, 220–226 (2013). 23273605 10.1016/j.jmoldx.2012.10.002 PMC5707183

[b7] Kudchadkar R., Paraiso K. H. & Smalley K. S. Targeting mutant BRAF in melanoma: current status and future development of combination therapy strategies. Cancer J 18, 124–131 (2012). 22453012 10.1097/PPO.0b013e31824b436e PMC3314865

[b8] Arkenau H. T., Kefford R. & Long G. V. Targeting BRAF for patients with melanoma. Br J Cancer 104, 392–398 (2011). 21139585 10.1038/sj.bjc.6606030 PMC3049553

[b9] Long GV1 W. J., Capper D., Preusser M., Zhang Y. E., Thompson J. F., Kefford R. F., von Deimling A. & Scolyer R. A. Immunohistochemistry is highly sensitive and specific for the detection of V600E BRAF mutation in melanoma. Am J Surg Pathol 37, 61–65 (2013). 23026937 10.1097/PAS.0b013e31826485c0

[b10] Richter A. *et al.* A multisite blinded study for the detection of BRAF mutations in formalin-fixed, paraffin-embedded malignant melanoma. Sci Rep 3, 1659 (2013). 23584600 10.1038/srep01659 PMC3625889

[b11] Siena S., Sartore-Bianchi A., Di Nicolantonio F., Balfour J. & Bardelli A. Biomarkers predicting clinical outcome of epidermal growth factor receptor-targeted therapy in metastatic colorectal cancer. J Natl Cancer Inst 101, 1308–1324 (2009). 19738166 10.1093/jnci/djp280 PMC2758310

[b12] Weisenberger D. J. *et al.* CpG island methylator phenotype underlies sporadic microsatellite instability and is tightly associated with BRAF mutation in colorectal cancer. Nat Genet 38, 787–793 (2006). 16804544 10.1038/ng1834

[b13] Markowitz S. D. & Bertagnolli M. M. Molecular origins of cancer: Molecular basis of colorectal cancer. N Engl J Med 361, 2449–2460 (2009). 20018966 10.1056/NEJMra0804588 PMC2843693

[b14] Xing M. BRAF mutation in thyroid cancer. Endocr Relat Cancer 12, 245–262 (2005). 15947100 10.1677/erc.1.0978

[b15] de Biase D. *et al.* High-sensitivity BRAF mutation analysis: BRAF V600E is acquired early during tumor development but is heterogeneously distributed in a subset of papillary thyroid carcinomas. J Clin Endocrinol Metab 99, E1530–1538 (2014). 24780046 10.1210/jc.2013-4389

[b16] Mathur A. *et al.* Higher rate of BRAF mutation in papillary thyroid cancer over time: a single-institution study. Cancer 117, 4390–4395 (2011). 21412762 10.1002/cncr.26072 PMC3131457

[b17] Adackapara C. A., Sholl L. M., Barletta J. A. & Hornick J. L. Immunohistochemistry using the BRAF V600E mutation-specific monoclonal antibody VE1 is not a useful surrogate for genotyping in colorectal adenocarcinoma. Histopathology 63, 187–193 (2013). 23763264 10.1111/his.12154

[b18] Day F. *et al.* A mutant BRAF V600E-specific immunohistochemical assay: correlation with molecular mutation status and clinical outcome in colorectal cancer. Target Oncol (2014) 10.1007/s11523-014-0319-8. [Epub ahead of print] PMC4363480 24859797

[b19] Boursault L. *et al.* Tumor homogeneity between primary and metastatic sites for BRAF status in metastatic melanoma determined by immunohistochemical and molecular testing. PLoS One 8, e70826 (2013). 23976959 10.1371/journal.pone.0070826 PMC3748080

[b20] Ilie M. I. *et al.* Diagnostic value of immunohistochemistry for the detection of the BRAF(V600E) mutation in papillary thyroid carcinoma: comparative analysis with three DNA-based assays. Thyroid 24, 858–866 (2014). 24417277 10.1089/thy.2013.0302

[b21] Angulo B., Lopez-Rios F. & Gonzalez D. A new generation of companion diagnostics: cobas BRAF, KRAS and EGFR mutation detection tests. Expert Rev Mol Diagn 14, 517–524 (2014). 24844134 10.1586/14737159.2014.910120

[b22] Colomba E. *et al.* Detection of BRAF p.V600E mutations in melanomas: comparison of four methods argues for sequential use of immunohistochemistry and pyrosequencing. J Mol Diagn 15, 94–100 (2013). 23159108 10.1016/j.jmoldx.2012.09.001

[b23] Chen Q. J. *et al.* Immunohistochemistry as a quick screening method for clinical detection of BRAF(V600E) mutation in melanoma patients. Tumour Biol 35, 5727–5733 (2014). 24563339 10.1007/s13277-014-1759-6

[b24] Lasota J. *et al.* Detection of the BRAF V600E mutation in colon carcinoma: critical evaluation of the imunohistochemical approach. Am J Surg Pathol 38, 1235–1241 (2014). 24832158 10.1097/PAS.0000000000000229 PMC4134735

